# Sniffer dogs performance is stable over time in detecting COVID-19 positive samples and agrees with the rapid antigen test in the field

**DOI:** 10.1038/s41598-023-30897-1

**Published:** 2023-03-05

**Authors:** Federica Pirrone, Patrizia Piotti, Massimo Galli, Roberto Gasparri, Aldo La Spina, Lorenzo Spaggiari, Mariangela Albertini

**Affiliations:** 1 Department of Veterinary Medicine and Animal Sciences, University of Milan (UNIMI), 26900 Lodi, Italy; 2 III Infectious Diseases Unit, L. Sacco Hospital, ASST Fatebenefratelli-Sacco, 20157 Milan, Italy; 3 Department of Biomedical and Clinical Sciences DIBIC, Luigi Sacco, University of Milan, Milan, Italy; 4 Division of Thoracic Surgery, IEO, European Institute of Oncology IRCCS, Milan, Italy; 5 Medical Detection Dogs Italy, Novate milanese, Italy; 6 Department of Oncology and Hemato-Oncology–DIPO, University of Milan, Milan, Italy

**Keywords:** Animal behaviour, Public health

## Abstract

Rapid antigen diagnostic (RAD) tests have been developed for the identification of the SARS-CoV-2 infection. However, they require nasopharyngeal or nasal swab, which is invasive, uncomfortable, and aerosolising. The use of saliva test was also proposed but has not yet been validated. Trained dogs may efficiently smell the presence of SARS-CoV-2 in biological samples of infected people, but further validation is needed both in laboratory and in field. The present study aimed to (1) assess and validate the stability over a specific time period of COVID-19 detection in humans’ armpit sweat by trained dogs thanks to a double-blind laboratory test–retest design, and (2) assess this ability when sniffing people directly. Dogs were not trained to discriminate against other infections. For all dogs (n. 3), the laboratory test on 360 samples yielded 93% sensitivity and 99% specificity, an 88% agreement with the Rt-PCR, and a moderate to strong test–retest correlation. When sniffing people directly (n. 97), dogs’ (n. 5) overall sensitivity (89%) and specificity (95%) were significantly above chance level. An almost perfect agreement with RAD results was found (kappa 0.83, SE 0.05, p = 0.001). Therefore, sniffer dogs met appropriate criteria (e.g., repeatability) and WHO's target product profiles for COVID-19 diagnostics and produced very promising results in laboratory and field settings, respectively. These findings support the idea that biodetection dogs could help reduce the spread of the virus in high-risk environments, including airports, schools, and public transport.

## Introduction

In the past 2 years, great efforts have been made to contain the COVID-19 pandemic worldwide. Due to high contagiousness of the severe acute respiratory syndrome coronavirus 2 (SARS-CoV-2), the resultant infection spread extremely fast and widely, provoking a sudden and substantial increase in hospitalisation of patients, due to pneumonia with multiorgan disease^1^. Implementation of large scale programs for virus detection have been essential to contain this emerging disease but represented a challenge to many countries, especially low- and middle-income ones^2,3^. Therefore, the urgency to develop diagnostic methods for SARS-CoV-2 that were faster, cheaper, and more accessible—but equally reliable—than the current gold standard, i.e. reverse transcription polymerase chain reaction–based (RT-PCR) immediately became clear^4,5^. As with many other infectious diseases, rapid antigen diagnostic (RAD) testing for COVID-19 is considered key to guiding social distancing orders and controlling disease clusters by contact tracing and isolation^6^. Nowadays, RADs are readily available to the public and can provide results within a very short time (e.g., 15 min)^7^.

However, implementing conventional rapid diagnostic tests has common potential pitfalls, as sensitivity and specificity may be lower than established laboratory testing (i.e. RT‐PCR)^8^, therefore requiring that these can only be used when the pre-test probability is high. In addition, quality control of testing can be difficult^9^ due to a variety of factors, including difficulty for most people to get a quality clinical specimen, particularly if the swab themselves, and the fact of RADs being approved as Clinical Laboratory Improvement Amendments of 1988 waived tests, which means that they are the least regulated and have the fewest quality control requirements^10^.

Based on the assumption that, similarly to a variety of other pathologies, including viral infections^11^, also COVID-19 could produce a unique pattern of volatile organic compounds (VOCs), several laboratories around the world have directed their efforts to train dogs to detect SARS-CoV-2-specifc VOC odour signature in biological samples from infected patients^12^. Olfaction is a dog’s primary special sense^13^, due to several physical characteristics: a uniquely extended olfactory cortex and area of the olfactory epithelium in the nasal cavity; nostrils shaped so as to enable sufficient odour molecules in the air flow to enter the nasal cavity; an extremely high number of olfactory receptors; a favourable proportion of active/inactive genes of the olfactory receptor proteins^14^. There is now evidence that dogs may indeed play a critical role in detecting human diseases, such as cancers^14,15^, showing comparable accuracy to that of standard diagnostic methods (i.e., gas chromatography and mass spectrometry analyses)^16^. Because they provide a real-time yes/no response to a detected odour (e.g., sitting or lying down), sniffer dogs have some notable advantages over VOCs detection technological devices. Being their binary response extremely rapid and clear, interpretation of the results is immediate and straightforward^17^. Therefore, although still requiring training and handling of dogs, this method may help avoid the need for personnel trained to process samples, as dogs sniff people directly, and prevent delays in issuing the test’s outcomes, as in the case of mass spectrometry.

The results of studies published so far on the accuracy of canine smell in detecting the presence of SARS-CoV-2 in biological samples (e.g., saliva, sweat, urine, trachea-bronchial secretions) from infected people suggest that sniffer dogs might reach percentages of sensitivity and specificity comparable to, or perhaps even higher^18^, than those of RT-PCR^3,4,19–23^. However, although most of these studies are of good quality, none of them provided scientific validation of canine scent detection, despite this being an important requirement in the chemical analysis practice^24^. Therefore, further applied research in this field is absolutely justified to provide definitive validation of this biodetection method.

Given the previous considerations, once dogs’ ability to detect COVID-19 from biological samples of positive people has undergone laboratory validation, for sniffer dogs’ to be successful in contributing to the sustainable control of the COVID-19 pandemic, they should be brought beyond lab testing and into the field. Scientists suggest that dogs could help control the pandemic because they could screen hundreds of people per hour in crowded places, such as airports or sports stadiums^25^, cheaply and in a non-invasive manner. However, so far, scientifical testing in real life contexts are still severely underrepresented^26^. Only one study has been formally published^27^ in which dogs were required to scent-interrogate the human body, reporting a decline in dog performance with prevalence, which however could be overcome improving the training method. Moreover, this study was conducted in Colombia in a non-vaccinated population, so it does not shed light on whether the COVID-19-vaccine affect the dogs’ ability to detect active infection.

The aims of the present study were to (1) assess reliability depending on sensitivity (Se) and specificity (Sp) of purposely trained dogs to indicate an odour associated with COVID-19 in human armpit sweat through a double-blind laboratory test–retest design, and (2) test trained dogs’ screening ability to detect people infected by SARS-CoV-2 by sniffing them directly in public spaces, comparing a scent detection dog test and nasopharyngeal SARS-CoV-2 RAD immunoassays. Community pharmacies and their incoming clients were chosen as settings for the screening in the field (aim 2) because, since Italian Cabinet of Ministers had declared a state of emergency on 31 January 2020^28^, pharmacies acquired an increasingly important role^29^ in the anti-epidemic response, which included the screening of potential cases of COVID-19 through RAD^30^.

## Results

### Phase 1: Laboratory testing

Data regarding the dogs’ response and the main characteristics of all the samples utilised during both the test and retest phases are reported as supplementary material (Supplementary Table 1). Volunteers had a median age of 51 years old (18–95 years, minimum–maximum), 63% were females and 77% had received anti-COVID-19 vaccination.

The combined sensitivity and specificity for all three dogs during the test phase was Se = 93% (95% CI = 84–103%) and Sp = 99% (95% CI = 97–101%). The combined sensitivity and specificity for all three dogs during the retest phase was Se = 83% (95% CI = 69–97%) and Sp = 97% (95% CI = 94–99%). Overall and individual Se and Sp are reported in Table 1. Based on the present prevalence of COVID-19 (17%), both the positive and negative predictive values were identical to Se and Sp, thus they will not be reported here for each individual dog. PPVs and NPVs were calculated according to this study prevalence (17%) and two hypothetical, opposite-strength prevalence (high and low) and are reported in Table 2.

**Table 1:** Canine ability to detect COVID-19-related VOCs in the laboratory test and retest.

Dog	Parameter	Test			Retest		
		Estimate	95% CI		Estimate	95% CI	
			Lower (%)	Upper (%)		Lower (%)	Upper (%)
Helix	Sensitivity	100% (10/10)	100	100	60% (6/10)	23	96
	Specificity	100% (50/50)	100	100	92% (46/50)	84	99
Otto	Sensitivity	90% (9/10)	67	112	90% (9/10)	67	112
	Specificity	98% (49/50)	93	102	98% (49/50)	93	102
Nala	Sensitivity	90% (9/10)	67	112	100% (10/10)	100	100
	Specificity	98% (49/50)	93	102	100% (50/50)	100	100

**Table 2:** Measured and simulated overall positive predictive values (PPVs) and negative predictive values (NPVs) with 95% confidence intervals.

Prevalence	Parameter	Estimate	95% CI	
			Lower	Upper
		Test		
17%	PPV	93%	78%	98%
	NPV	99%	95%	100%
40%	PPV	98%	92%	99%
	NPV	96%	85%	99%
3%	PPV	68%	35%	90%
	NPV	100%	99%	100%

Overall, discrepancies between the dogs’ response and the RT-PCR result were observed for 14/360 samples (3.9%), 50% of which were positive. Seventy-one per cent (n = 10) of the discrepancies were found in the retest (Table 3), all but two to be attributed to one dog (Helix).

**Table 3:** Main biological characteristics of samples and experimental phase (test/retest) associated to discrepancies between SARS-CoV-2 RT-PCR and the response from the three dogs.

Dog	Sample id	RT-PCR diagnosis	Phase	Participant gender	Participant age (yrs)	Vaccination
Otto	P_129	Positive	Test	F	87	Yes
Nala	P_121	Positive	Test	F	74	No
Otto	I187	Negative	Test	F	50	Yes
Nala	I170	Negative	Test	F	66	Yes
Helix	P_121	Positive	Retest	F	74	No
Helix	P_139	Positive	Retest	M	85	Yes
Helix	P_113	Positive	Retest	F	69	Yes
Helix	L233	Positive	Retest	F	29	No
Otto	P_151	Positive	Retest	M	61	Yes
Helix	I99	Negative	Retest	M	61	Yes
Helix	L274	Negative	Retest	F	38	Yes
Helix	L75	Negative	Retest	M	60	No
Helix	L174	Negative	Retest	F	32	Yes
Otto	I382	Negative	Retest	F	31	Yes

As for test–retest reliability overall, an 88% agreement was found between test and retest, which refers to the percentage of trials for which there was no discrepancy between the two phases. Regarding the Spearman’s correlation, one dog (Nala) yielded a Spearman’s r = 1 (100% correct responses). However, the percentage agreement for Nala was 91%. When considering the other two dogs individually, a moderate to strong test–retest reliability was found for Otto (90% agreement, Spearman’s r = 0.7, p = 0.035, 95% CI 0.042–0.917) while the reliability was lower for Helix (83% agreement, Spearman’s r = 0.5, p = 0.082, CI − 0.093 to 0.849).

As for GzLM model results, none of the factors included was a significant predictor for the dogs’ correct response (all p > 0,05, data non shown).

### Phase 2: in field work

In total, 97 volunteers (54% women) underwent both sniffer dog and RAD test (results are reported in Tables 4, 5 and in supplementary Tables 2 and 3). Thirty-eight of them were investigated by more than one dog.

**Table 4:** Summary of results from volunteers who tested positive on the rapid antigen test (RAD) and were sniffed by the sniffer dogs in the field work, grouped based on being sniffed by a single dog (one) or multiple dogs (multiple).

Number of sniffer dogs examining each volunteer	Dog	Correct choice rates
One	HOPE	n. correct choices/total positive = 82% (13/16) n. incorrect choices/total positive = 18% (3/16)
One	IRIS	n. correct choices/total positive = 100% (1/1) n. incorrect choices/total positive = 0% (0/1)
One	NIM	n. correct choices/total positive = 100% (10/10) n. incorrect choices/total positive = 0% (0/10)
One	CHAOS	n. correct choices/total positive = 100% (1/1) n. incorrect choices/total positive = 0% (0/1)

Volunteer code	Number of sniffer dogs examining each volunteer	Correct sniffer dog
dmmau	Multiple	NIM
		HOPE
dsgua	Multiple	NIM
		HOPE
drsof	Multiple	HOPE
		IRIS
		NIM
		CHAOS
dae	Multiple	NALA
		HOPE
		NIM
drb	Multiple	NALA
		HOPE
dem	Multiple	NALA
		IRIS
		NIM
dgm	Multiple	NALA
		IRIS
dibna	Multiple	HOPE
		IRIS
dpessa	Multiple	IRIS
		CHAOS
fnid2	Multiple	**NALA**
		**HOPE**
fnif	Multiple	HOPE
		**NIM**

Names of sniffer dogs with wrong choices are displayed in bold.

**Table 5:** Summary of results from volunteers who tested negative on the rapid antigen test (RAD) and were sniffed by the sniffer dogs in the field work, grouped based on being sniffed by a single dog (one) or multiple dogs (multiple).

Number of sniffer dogs examining each volunteer	Dog	Correct choice rates
One	NALA	n. correct choices/total negative = 100% (2/2) n. incorrect choices/total negative = 0% (0/2)
One	HOPE	n. correct choices/total negative = 95% (20/21) n. incorrect choices/total negative = 5% (1/21)
One	NIM	n. correct choices/total negative = 100% (7/7) n. incorrect choices/total negative = 0% (0/7)
One	CHAOS	n. correct choices/total negative = 100% (1/1) n. incorrect choices/total negative = 0% (0/1)

Volunteer code	Number of sniffer dogs examining each volunteer	Correct sniffer dog
dqa	Multiple	NALA
		IRIS
		NIM
		CHAOS
		HOPE
dalb	Multiple	NALA
		HOPE
fnifu	Multiple	NALA
		HOPE
fnifd	Multiple	NALA
		HOPE
drev	Multiple	HOPE
		IRIS
		NIM
		CHAOS
dce	Multiple	NIM
		NALA
		HOPE
dpl	Multiple	NALA
		HOPE
		NIM
fnirzo2	Multiple	HOPE
		NALA
fnirza	Multiple	NALA
		HOPE
dgr	Multiple	IRIS
		CHAOS
dmr	Multiple	IRIS
		CHAOS
dcg	Multiple	IRIS
		CHAOS
dsa	Multiple	IRIS
		CHAOS
fnid3	Multiple	NALA
		HOPE
fnid4	Multiple	NALA
		HOPE
Fnim	Multiple	**NIM**
		HOPE
fnid11	Multiple	**HOPE**
		**NIM**
Ftu	Multiple	NALA
		HOPE
dpa	Multiple	NALA
		HOPE
		NIM
fmabno2	Multiple	HOPE
		IRIS
fmad	Multiple	HOPE
		IRIS
fmanza2	Multiple	HOPE
		**IRIS**
fmanzo2	Multiple	HOPE
		IRIS
fmarzo	Multiple	HOPE
		IRIS
fnid	Multiple	NALA
		HOPE
fnirza2	Multiple	NALA
		HOPE
ftrzo	Multiple	NALA
		HOPE

Name of sniffer dogs with wrong choices are in bold.

The rapid antigen test gave a positive result in 39 volunteers (40%) (Table 4, supplementary Table 2). Twenty-eight of them were investigated by one single dog among Hope, Nim, Iris and Chaos. For three positive, but asymptomatic, cases the sniffer dog response diverged from the antigen test result. The remaining 11 volunteers were sniffed by 2 or more dogs: for 9 of them, 6 of whom were asymptomatic, all dogs signalled consistently with the RAD; one positive volunteer, referring weak symptoms, was smelled by 2 dogs, both signalling divergently from the antigen test result, e.g., as if he was negative, while another volunteer was smelled by 2 dogs, only one of whom signalled consistently with the RAD.

Of the 58 (60%) volunteers tested negative on rapid antigen test (Table 5, supplementary Table 3), 31 were investigated by one single dog among Nala, Hope, Nim and Chaos, with only one indicating divergently from the RAD, e.g., signalling as if the volunteer was positive instead of ignoring them. The remaining twenty-seven were sniffed by 2 or more dogs: on 24 of them, all the dogs’ signalling was consistent with the antigen test result (i.e., negative); one participant complaining COVID-19-like symptoms but receiving a negative diagnosis was smelled by two dogs, both signalling divergently from the antigen test result (e.g., as if he was positive); finally, in two different cases, two dogs diverged from each other in their response.

Regarding the overall Se and Sp of sniffer dogs working in field, the Se was 89% (95% CI 78–95%) and the Sp was 95% (95% CI 88–98%). Thus, the 95% CI did not overlap by 50%, being far from randomness.

Overall, there was an almost perfect concordance between the dogs’ detection and RAD results (kappa 0.84, se 0.047, p = 0.001).

## Discussion

In the present study, firstly, three family dogs were purposely trained to detect COVID-19 related VOCs in human armpit sweat.

### Double-blinded test–retest reliability assessment in laboratory

The prime contribution of the current paper lies in answering the empirical question of whether trained sniffer dogs’ ability is relatively stable over time in a laboratory setting. The current results support such possibility, at least in two of the three dogs involved. The dogs’ ability was analysed through test–retest reliability, in order to verify whether their performance achieved the same results when repeated after a short period of time. According to the results, Otto and Nala had high Se and Sp, which remained high, or even increased in the case of Nala, between the test and the retest. By contrast, Helix had the highest test’s Se and Sp (100%) but was markedly less successful during the retest. It could be useful to report that*,* between the test and the retest, both of Helix’s owners, who she lived with, contracted COVID-19 which would lead one to consider that Helix may have been in persistent contact with the smell of the virus. Thus, the possibility that this phenomenon may have confused Helix, or that she had acclimatised to the smell, cannot be excluded. Although this is too weak to be put forward as one possible explanation for decreasing of her detection performance it emerges as an aspect to consider in the management of sniffer dogs who are involved in this type of activity. It is worth noting that, such as neuroimaging measurements, other authors defined less lenient values for test–retest correlation than those considered in the present study. For example, Portney and Watkins^31^ proposed values between 0.5 and 0.75 as “poor to moderate”, 0.75 to 0.9 as “good”, and above 0.9 as acceptable for clinical measures. However, these higher standards cannot be considered to be directly applicable to conditions similar to those reported here^32^ because they are defined considering that in clinical measurements, where physiological parameters are estimated, within-subject fluctuations can usually be assumed to be negligibly small over short time periods. In the present study, COVID-19-related VOCS are expected to be stable in all positive samples, and the aim was precisely to validate the dogs’ retention, in a short period of time, of the ability to detect the COVID-19 VOCS’s signature. However, so we cannot completely exclude the potential effect of the volunteers’ different identity on the reliability estimate. More conservatively, this condition could possibly resemble that of psychometric questionnaires test–retest reliability^33,34^, with reliability coefficients necessarily being lower, because they include both measurement error within the rater and biases due to actual changes in the underlying true value^32,35^.

During the laboratory test, the three trained dogs showed an overall sensitivity rate of 93% (90–100%), and an overall specificity rate of 99%, which ranged from 98 to 100%. These values fall within the upper end of published ranges. In fact, in studies conducted recently on olfactory detection of COVID-19 in sweat samples by sniffer dogs^3,22,23,36^, the sniffer dogs’ sensitivity ranged from 68 to 100% and the specificity ranged from 75 to 91%. Moreover, laboratory test Se and Sp recorded in the current study were well above the minimum performance criteria set by World Health Organization (WHO) for SARS-CoV-2 antigen-detecting rapid diagnostic tests (≥ 80% sensitivity and ≥ 97% specificity) and for antibody test kits (> 90% sensitivity and > 95% specificity), being also very close to those set for RT-PCR based test kits (≥ 95% sensitivity and ≥ 99% specificity)^37^. Helix’s high sensitivity and specificity indicate that her rate of false-negative and false-positive results was low, which, if confirmed in the field, would support the usefulness of sniffer dogs either for large-scale screening or diagnosis confirming^23^. Moreover, the dogs showed to be highly sensitive and specific despite being presented with samples from participants recruited from multiple and different sampling sites, either hospitalised or not, which one may expect to increase the risk of errors induced by diverse background odours^23^. When controlling for the impact of participants’ potential confounders, including gender and vaccination status, no significant effects were found on the dogs’ diagnostic abilities. Although the lack of effect of the gender could be expect based on previous studies^22^, the lack of effect of a participant’s vaccination status was surprising, given that Devillier et al.^23^ reported decreased Se and increased Sp, and a nearly halved PPV, in sweat samples from 76 vaccinated individuals. Considering that vaccination is so far extremely common in the general population, with more than 11.9 billion doses been administered across 184 countries^38^, our result provides further strengthening of the potential use of sniffer dogs for mass screening. In the present study we did not test for the effect of symptoms, so the potential confounding effect of symptoms of non-COVID-19 conditions cannot be excluded.

The high sensitivity and specificity and low inter-dog difference observed in the present study likely originate from two conditions: (1) the “ubiquitous” recruitment system and the rigorous sample collection, and (2) the consistent high-quality training performed.

In our study, positive and negative samples were collected from both hospitals and outside hospitals, symptomatic and asymptomatic volunteers, vaccinated and non-vaccinated, so that the actual target population could be accurately covered and potential misleading of dogs due to hospital-associated odours avoided. In addition, as recommended^14,22^, in order to mimic better a real life situation, particularly under low prevalence conditions, we randomly included trials with only negative samples.

Following the example of Kantele et al.^22^, based on the current study overall Se and Sp (Se = 93% and Sp = 99%, respectively), PPV and NPV were calculated according to two scenarios reflecting different prevalence of infection (high prevalence = 40% and low prevalence = 3%). For populations with a prevalence of 40%, such as hospitals or nursing homes during the pandemic^39^, we obtained a PPV of 98% and a NPV of 96%. This means that the response provided by the dogs would increase by 5% the probability that a sample from the Case group was indicated by the dogs as positive, approaching a 100% Se. However, the true COVID-19 prevalence estimate is currently thought to be quite low in many areas (3% on average)^40^, particularly in public settings like airports or schools^41^: for such populations, we estimated a PPV of 68% and an NPV of 100%. This indicates that the dogs’ response would increase the chances for a sample from the Control group being indicated by the dogs as negative to 100%. Assuming that highly sensitive tests are best used for screening, while highly specific tests are best for confirming diagnostics^22^, the high PPV and NPV achieved in the two estimated prevalence scenarios support the potential use of dogs both in sites of high SARS-CoV-2 prevalence to exclude individuals not needing RT-PCR, and in low prevalence sites to complement the standard quick diagnostic test in confirming that the virus is present^42^. Clearly, if a COVID-19 diagnosis is missed, this can lead to delayed, missed, or unneeded treatment for an inaccurate alternative diagnosis.

### In field training procedure

As for phase 2, the present study is the first evaluating trained sniffer dogs as point-of-care test for SARS-CoV-2 in one of the types of public facility most prone to overload, the community pharmacies. Since late 2020, in Italy, the demand for COVID-19 testing has quickly transformed the pharmacies in points-of-care. Approximately 70% of the 20.000 pharmacies across Italy were offering swabs at the time of our study, performing hundreds of swabs per day, especially amid variant surges. Their involvement indeed caused a 80% increase in the country administration of swabs^43^. Unfortunately, despite this increase in testing activity, fair access to testing remained a challenge^44^. Trained sniffer dogs represent good candidates for point-of-care and mass surveillance testing to rapidly detect SARS-CoV-2-infected humans, counterbalancing a sensitivity that is lower than the gold standard molecular tests with fast results and the possibility of repeated testing. According to D’Aniello et al.^26^, the major problem COVID-19 scent-work is that very few data are available on the performance of dogs in the field, as the application of the models in the laboratory studies has not been scientifically tested in real life contexts. The present study aimed to contribute to filling this gap, thus clarifying the effectiveness of dogs in this area. When sniffing people directly, the dogs’ overall sensitivity and specificity were high. In particular, the dogs reached 89% sensitivity, that was way above the minimum requested by WHO for SARS-CoV-2 RAD (≥ 80%); additionally, the kappa coefficient for field tests revealed an almost perfect agreement with RAD results. Thus, the canine performance as screening test to correctly identify positive people was satisfactory and comparable to that of a standard screening swab test.

There was a difference between the duration of training of dogs in laboratory settings and the time needed to train the other five dogs in field. This was shorter compared to the duration of laboratory training for Nala and Otto, which can be explained by a different individual attitude of the dogs and by the fact that working in a laboratory setting, on samples contained in stainless steel supports, requires a longer time for learning than working in conditions that are way more natural to the dogs, namely outdoors, in public places to which they are already used, in a task that consists in sniffing out people. For Helix, the duration of lab training was shorter compared to Nala and Otto, which can be explained by taking into account that, as we explained in the methods section, she was already engaged in bio-detection work with human lung cancer. That work was carried out in the same lab, with the same scientists: therefore, her training did not require time to get used to the environment, but only to the new smell and to the sample supports, that in this study were aluminium tracks positioned on the ground, while in the lung cancer study they were 60-cm high aluminium stands.

The Sp reached by our five dogs in the field (94%) was disappointingly lower than the 97% threshold set by the WHO for validation of antigen tests. Although such value could still be acceptable for screening^45^, determining the reason for the relatively low Sp could help improve it. As previously suggested^27^, odour contamination in a real life condition may have caused the reported suboptimal level of specificity, which could cause slightly more false positives than one would expect. In addition, some of the false positives referred to people with negative swab test results and COVID-19-like symptoms. Unfortunately, one can only speculate on this issue, because the experimental design did not include RT-PCR diagnosis confirmation for people enrolled in the current field test. Moreover, for ethical reasons the participants in the current study could provide us with information about the presence/absence of symptoms on a voluntary basis, and only a minority of them provided this information. According to the rules in place in Italy to minimise the spread of COVID-19, at the time when the study was performed, if someone suspected they might have COVID-19 based on their epidemiological status and their symptoms, they would have had to undergo isolation and lockdown. This probably contributed to discouraging volunteers from sharing COVID-19 health-related details. However, during testing of the four volunteers whose RAD was negative but were scored positive on sniffer dog testing, one declared symptoms consistent with COVID-19, i.e. respiratory symptoms. As observed by other authors^3^, it is possible that the dogs in our study were identifying other respiratory infections. However, dogs’ ability to discriminate samples from SARS-CoV-2 infected individuals from those infected with other pathogens had already been shown in previous studies^46,47^, which is the reason why, purposely, we chose not to control for this issue here. This would have required, for example, to train dogs including a third group volunteers with non-COVID-19 respiratory conditions and present at least one sample from this group in each run.

## Conclusions

In conclusion, the sniffer dogs involved in the current study demonstrated the foundations of a good screening test: cause minimal discomfort to the human volunteers, be inexpensive to perform, easy to administer, reliable and valid in discriminating diseased and non-diseased humans. Therefore, they may be potentially useful for mass detection and contact tracing for COVID-19, being implemented in various public settings, such as schools or airports, where conventional testing is not feasible or easily accessible. Moreover, the potential usefulness of dogs could be extended to other potential health screening applications. Although, after 4 years, COVID-19 continues circulating, driven by the wildly transmissible new variants, these findings suggest that dogs could be trained and deployed as a first response to new threats or future pandemics.

Sniffer dogs performed better than RAD under controlled settings but were similar to RAD in terms of sensitivity in field conditions. However, compared to RAD, sniffing dogs hold some relevant advantages: for example, they do not impose unpleasant procedures, as the nasopharyngeal/nasal swabs, thar are commonly perceived to be uncomfortable, or even painful; they provide timely identification and isolation of the largest number of patients in real-life contexts, thus improving access to communities and simplifying logistics; and do not require operators to come into direct contact with potentially infectious materials. Further efforts seeking standardisation and validation of the processes are needed to better understand the potential and limitation of using sniffing dogs for the detection of COVID-19 and verify them in appropriate populations. It is worth noticing that the consistency in error rates for positive early-stage COVID-19 between the laboratory test and in-field test was potentially the source of the increased error rates in in-field test, in addition to the change from the sweat sample to the entire body odours including clothes, cosmetics, and so on. In our study, training with diverse positive and negative volunteers, we aimed to improve the correct rates in COVID-19 detection by the sniffer dogs. Nevertheless, further research should include more high quality exploration of the influence on the odour signature (or the perception of it) by variables such as training sample number and type, sampling method, dogs’ personalities^48^, as well as of dogs’ performance in in field scenarios.

## Methods

The study was approved both by the University of Milan Ethical Committee (CE_26_21 and CE 84/21) and the Institutional Review Board of the European Institute of Oncology (R90/14-IEO102), in accordance with the relevant guidelines and regulations, and individual written informed consent was obtained from all the study participants after appropriate information concerning the study was provided. Eligible participants were people of either gender, older than 7 years; in the case of minors, written informed consent was provided by a legal tutor. Participation to provide a sweat sample or to be sniffed by trained dogs was volunteer. Anti-COVID-19 vaccination was not an exclusion criterium. Whenever possible, the type, number of dose and date of vaccine administration were also recorded after the information was provided on a voluntary basis.)

### Phase 1: Laboratory training and testing

The first phase of the study consisted of training dogs to discriminate in the laboratory between sweat samples from patients with COVID-19 and sweat samples from healthy controls.

#### Participants and sample sourcing

The samples were collected from: the III Infectious Diseases Unit, L. Sacco Hospital, ASST Fatebenefratelli-Sacco, Milan, Italy; the Division of Thoracic Surgery of the European Institute of Oncology (IEO), Milan, Italy; COVID-19 screening stations across the North of Italy.

Participants were divided into two groups as shown in Table 6:

**Table 6:** Groups and sample size of training and test–retest phases.

Group	Description	Phase		
		Training	Test	Retest
CASES	Patients COVID-19 positive on RT-PCR for SARS-CoV-2	103	24	19
	Positive non vaccinated	87	16	5
	Positive vaccinated	16	8	14
CONTROLS	Patients COVID-19 negative on RT-PCR for SARS-CoV-2	338	124	109
	Negative non vaccinated	102	28	18
	Negative vaccinated	236	96	91

1. Cases, N. 146 patients COVID-19 positive, who had tested positive by RT-PCR for SARS-CoV-2 within 24 h prior collection, regardless of COVID-19 symptoms.
2. Controls, N. 571 patients COVID-19 negative, who had tested negative by RT-PCR for SARS-CoV-2 within 24 h prior collection.

The number of samples was calculated based on the number of training and test–retest sessions required according to the training technique we developed, so that dogs could be presented with several samples while ensuring a case: control ratio of 1:5. Samples collected were used multiple times only during training.

#### Sample collection and handling

Armpit sweat samples were collected following the same standard protocol for all volunteers and using the same media across all locations. The volunteers were asked to hold an inert polymer tube (3.6 cm length, 0.8 cm thickness) commonly used for adsorbing VOCs for explosive, drug, or criminology detection (Getxent, Neuchâtel, Switzerland) simultaneously under each armpit for 20 min. Each individual polymer tube was immediately placed in a sealed envelope, bearing the subject's ID. The samples were then shipped while refrigerated (+ 4 to + 8 °C) to the laboratory of the dog training/testing centre, in separate packaging for cases and controls, and kept stored at a temperature of 4 °C. The choice to work with sweat samples was made, in accordance with what reported by Grandjean et al.^49^, because sweat does not appear to be an excretion route for SARS-CoV-2 virus and can be easily collected in a non-invasive way. It appears however to carry COVID-19 VOCs^49^. Moreover, sweat allows the anticipation of practical applications of trained dogs to detect SARS-CoV-2 virus on positive people.

#### Animals

Dogs were provided by local dog owners and screened for inclusion in the study. The initial screening phase included the assessment of the effectiveness of food as a reinforcer for each dog and the dog's suitability for work in a laboratory environment in the presence of non-familiar people. Out of the 6 dogs that met the inclusion criteria, 3 dogs underwent the entire training session, while the others were exempted, due to either health or management problems, or low motivation for working in the scent line-up. One 8 years old Belgian Malinois female dog (Nala), an 8-year-old female mixed-breed dog (Helix), and a 5-year Dachshund male dog (Otto) underwent a training period (two weekly training sessions) to discriminate between the sweat of people with COVID-19 and the healthy controls. The training lasted 5 months for Helix and 7 months for Nala and Otto. All dogs had previous experience with bio-detection work (Helix: human lung cancer^15^, Nala and Otto: phyto aromas and *Cimex lectularius*).

The dogs were not used for other tasks during the study. All dogs lived at home with their owners, they were handled by professional dog trainers or behaviour scientists during the training and testing sessions, and were cared for by their owners between sessions.

#### Sniffing room and experimental equipment

**Figure 1:**
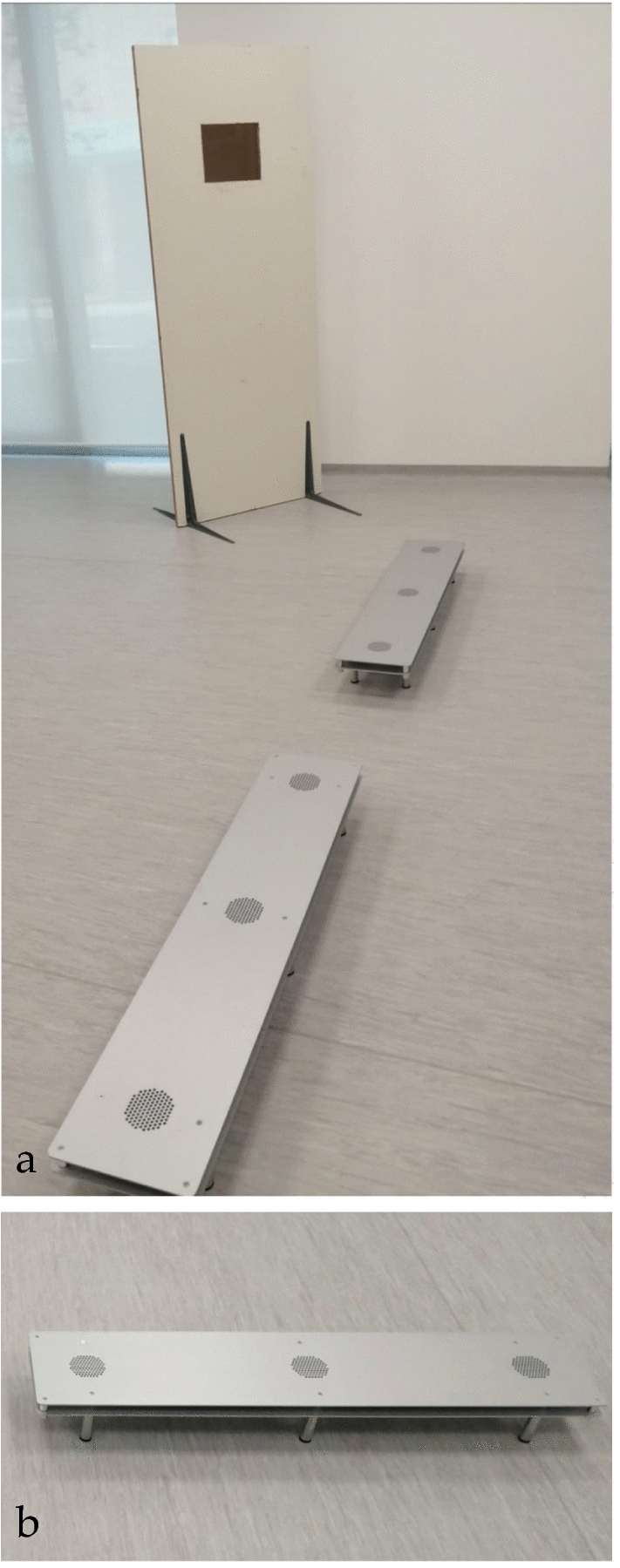
(**a**) The two especially designed stainless steel and aluminium apparatuses positioned in a single straight line on the floor, spaced 40 cm apart; (**b**) detail of the apparatus.

Dog training and testing was conducted at the laboratory of Animal PhysioEthology of the Department of Veterinary Medicine and Animal Sciences, University of Milan, Lodi, Italy. Two tracks of three samples, especially designed for this study, and constructed of aluminium, were positioned in a single straight line, spaced 40 cm apart, on the floor, as in Fig. 1. A sterile container with the sweat-stained cylinders was put into each dedicated holder, which was protected by a perforated metal cover, for the dogs to sniff. The cylinder was not visible or accessible to the dogs other than by olfaction. For self-protection and to prevent cross-contamination, the study team wore powder-free nitrile gloves and masks when handling the containers and sample cylinders.

#### Training procedure

**Figure 2:**
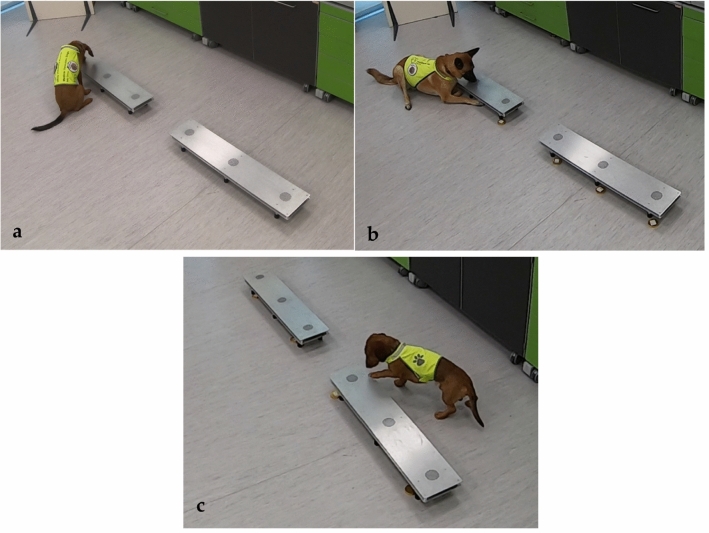
Helix (**a**) Nala (**b**) and Otto (**c**) indicate the correct position respectively by sitting, lying, and scratching directly in front of/on the sample station.

The training aimed to teach the dogs to discriminate Case and Control sweats and indicate the cases. The training procedure was carried out by Medical Detection Dogs Italy’s bio-detection trainers and was based on operant conditioning, with a food reward provided for correct behaviour as described previously^15^. Briefly, two test days per week were scheduled, each with two sessions of at least five trials, depending on the dog's level of motivation. In each trial, samples were selected to fill the 2 three-slot tracks so that one sample from the Case group was compared with five samples from the Control group, always varying the positive (Case) donor's identity. To minimise position-related interference, the location of each sample was randomly changed throughout the trials. At the end of each session, the slots were changed and the sample tracks were thoroughly cleaned with a vapor machine (Vaporetto PRO 90 Turbo, Polti, Italy). On each trial, the dogs were required to indicate the positive samples by sitting, lying, or scratching directly in front of the holder containing the COVID-19 sample (Fig. 2). In the last part of training, empty runs implying presentation of all negative samples were also performed. The training was considered completed when the dog was able to detect one positive sample out of six samples, as confirmed by achievement of a success rate ≥ 80% in two subsequent sessions of five trials. All dogs reached this threshold.

#### Double-blinded test–retest reliability assessment

The test and the re-test aimed to measure the accuracy of the training. Each dog was naïve to the samples they used during both the test and retest phases, meaning that they had never been presented before with one of those samples. However, more than one dog could be presented with the same sample from a given volunteer and dogs could sniff during the test or the re-test samples that another dog had used in training. For this reason, during these phases the three dogs were presented with a total of n. 360 unfamiliar samples (n. 300 negatives and n. 60 positives), who were collected from a total of n. 282 participants (n. 233 RT-PCR negative, n. 43 RT-PCR positive). During testing and re-testing, only the trainer was inside the room, and she remained hidden by the wooden screen (Fig. 1) while the dog performed the research work.

At the beginning of the test, before the dog and the trainer entered the room, one positive and five negative samples were selected and placed in the six holders by an experimenter, who left the laboratory soon after. The location of each sample was pre-determined by this experimenter in a pseudo-random order (designed using http://www.random.org), so that the positive sample was in a different location in each trial. A second experimenter was in an adjacent room, avoiding any interaction with the trainer and observing the dogs' work and behaviour through a digital video-camera (GoPro HERO7, Italy), which was mounted on a wall and operated remotely. The study was double-blinded setting, since the type and position of the samples in the sniffing stations were unknown to both the trainer and the second experimenter.

The test took place on 2 days, 1 week apart, on which every dog performed 1 session of 5 trials. For each trial, a new set of samples of volunteers was used. However, some of the samples might have been used during training by another dog. At the end of each trial, the dog was rewarded verbally regardless of whether the marking was correct or not.

To assess the repeatability (or test–retest reliability)^50^ of canine olfactory detection COVID-19 related VOCs in human sweat samples, a retest was conducted in two additional and successive sessions, 1 week apart, within 3 weeks after the second test session and under the same conditions. During this time, the dogs did not work in olfactory research.

### Phase 2: field study

#### Animals

Five dogs underwent the training process for the field study. One of these was Nala, the Belgian Malinois who was also involved in the laboratory testing phase. The other dogs were Chaos (Golden retriever, male, 4.5 years old), Hope (Border collie, female, 6 years old), Iris (Golden retriever, female, 3 years old) and Nim (mixed breed, female, 12 years old). Only Nala and Nim had previous experience of scent work (phyto aromas and *Cimex lectularius*). These dogs were recruited based on their behavioural characteristics, such as search ability and fearfulness.

#### In field training procedure

The trainers and the method (operant conditioning with positive reinforcement and a food reward provided for correct behaviour) employed were the same as in laboratory testing except they did not use the apparatuses to hold the samples. Training for the field screening was carried out from September 2021 to mid-January 2022, and passed through two steps, as described below, during which the dogs learnt to identify COVID-19 patients by sniffing the volunteers directly. Step one training sessions were conducted at various drive-thru COVID-19 testing points after volunteers had received their negative antigen test result. This was necessary so that, adding a positive scent or not, could reliably allow for presenting the dogs with positive and negative scents on humans. Round-shaped metallic stainless-steel boxes with 6 holes (height: 1.2 cm; diameter: 3.6 cm), typically employed in nose-work, were used during the training. The boxes contained either a positive or negative sweat-stained cylinder. The boxes used for positive samples were not mixed with the boxes used for negative samples. In each training session, the dogs had to enter a room where a maximum of 5 volunteers lined up having a box hidden in their clothing, preferring areas where people are expected to sweat the most (e.g., sleeves, shoes, etc.). The location of the person holding the positive sample was always randomised in the line-up. The volunteers could either be sitting or standing in a queue, thus simulating a typical situation in a testing point, including hospitals and pharmacies, and dogs were required to indicate that they recognised the scent of COVID-19 by stopping in front of the person holding the box with the target odour (positive scent). As in the training phase, the scent was obtained from patients with a diagnosis confirmed by RT-PCR. Overall, the dogs were trained using 131 positive samples. None of the samples presented to Nala (who had participated in phase 1) came from volunteers who had participated in the laboratory testing phase. This training step lasted 2 months for each dog.

In step 2 of the field study, 192 incoming clients at community pharmacies volunteered for the study. In each trial, the dogs could sniff any part of the body of one volunteer and they were allowed to touch with their noses the body of the volunteer. This training step lasted 2.5 months for each dog.

The trials in step 1 were unblinded (e.g., the handlers knew the position and number of the sniffers containing the positive cylinders) to observe the spontaneous behavioural cues offered by each dog when alerting on the target odour (positive scent). In step 2, the trials were single-blinded, as the handlers were unaware of who had received a positive diagnosis. By contrast, the volunteers and the director of training, who was present during both phases, were always unblinded, and the director informed the dog-trainer dyad of every correct alert. The results of this training step are reported in Table 7 and supplementary Table 5.

**Table 7:** Training of sniffer dogs for field work on volunteers.

DOG	Antigen test	Sniffer dog
NALA	n. total positive = 32	n. correct choices/total positive = 88% n. incorrect choices/total positive volunteers = 12%
	n. total negative = 56	n. correct choices/total negative = 98% n. incorrect choices/total positive volunteers = 2%
HOPE	n. total positive = 19	n. correct choices/total positive = 100% n. incorrect choices/total positive volunteers = 0%
	n. total negative = 33	n. correct choices/total negative = 91% n. incorrect choices/total positive volunteers = 9%
IRIS	n. total positive = 11	n. correct choices/total positive = 82% n. incorrect choices/total positive volunteers = 18%
	n. total negative = 21	n. correct choices/total negative = 100% n. incorrect choices/total positive volunteers = 0%
NIM	n. total positive = 1	n. correct choices/total positive = 100% n. incorrect choices/total positive volunteers = 0%
	n. total negative = 9	n. correct choices/total negative = 89% n. incorrect choices/total positive volunteers = 11%
CHAOS	n. total positive = 2	n. correct choices/total positive = 100% n. incorrect choices/total positive volunteers = 0%
	n. total negative = 8	n. correct choices/total negative = 100% n. incorrect choices/total positive volunteers = 0%

#### In field screening procedure

In mid-January 2022, in-field screening begun on human volunteers in North Italy’s pharmacy COVID-19 testing queues, in order to screen the general population. Sessions were carried out once a week, between February 2022 and May 2022. During each daily session, each dog was asked to investigate a maximum of 10 people, depending on their motivation. The director of training was always present as an external controller, ensuring that the predetermined protocol was followed. For each session, the tests were triple-blinded with the diagnosis being unknown to everyone involved (handler, director of training, and participants). As in the in field study by Vesga et al.^27^, the director had to interpret the behaviour of each dog to decide if a food reward was due. In the screening, the volunteers were sniffed out by a dog shortly after their COVID-19 rapid antigen test, and before receiving the results. Since we wanted to evaluate performance under real-life conditions, the dogs worked on leash as, in Italy, this is how they are required to be handled in all public areas.

#### Statistical analysis

Statistical analysis was performed using IBM SPSS Statistics for Windows, version 27.0 (Armonk, NY: IBM Corp).

As for phase 1 (laboratory testing), the sensitivity (Se) and specificity (Sp) of a dog's indication of samples compared with the true diagnosis confirmed by RT-PCR were calculated. The sensitivity refers to the conditional probability of the dog indicating COVID-19 when the condition was present, and specificity refers to the conditional probability of the dog ignoring a sample from a healthy donor. Both sensitivity and specificity were expressed as proportions. Point estimates were calculated with 95% confidence intervals. The probability of a perfect test trial (finding the right sample and ignoring the controls) by chance was 1/6 (17%). Similarly to a previous study^22^, positive predictive values (PPVs) and negative predictive values (NPVs) with 95% confidence intervals were also calculated based either on our prevalence or on a high (40%) and low (3%) hypothetical scenarios of prevalence^40,51^. PPVs and NPVs were obtained from Se, Sp, and prevalence according to the Bayes’ rule^52^:

$${\text{PPV}}\, = \,\left( {{\text{Se}}\, \times \,{\text{prevalence}}} \right)/\left[ {\left( {{\text{Se}}\, \times \,{\text{prevalence}}} \right)\, + \,\left(1 - {\text{Sp}} \right)\, \times \,\left( 1 - {\text{prevalence}} \right)} \right]$$

$${\text{NPV}}\, = \,{\text{Sp}}\, \times \,\left( 1 - {\text{prevalence}} \right)/\left[ {\left( 1 - {\text{Se}} \right)\, \times \,{\text{prevalence}}\, + \,{\text{Sp}}\, \times \,\left( 1 - {\text{prevalence}} \right)} \right].$$

Percentage of agreement^53^ and Spearman's correlation^35^ were computed to measure the degree to which dogs’ detection ability to detect COVID-19 was identical to the RT-PCR, and to establish temporal reliability, between the test and the retest, respectively. According to Schober et al.^54^, Spearman's correlation coefficient was interpreted as follows: 0.00–0.10 = negligible correlation, 0.10–0.39 = weak correlation, 0.40–0.69 = moderate correlation, 0.70–0.89 = strong correlation, and 0.90–1.00 = very strong correlation. A generalised linear model (GzLM) with binary response analysis was performed to identify either test- or volunteer-related factors (dog, RT-PCR diagnosis, testing phase, participant gender) influencing a dog's correct response. The dogs’ response (yes/no) was entered as dependent variable and all factors were measured for main effects. The strength of the associations was expressed as odds ratio (OR) and 95% confidence interval (95% CI).

As for phase 2 (field work), Se and Sp were calculated compared with the RAD result. In each trial, the probability of success or failure was 50%. Thus, we calculated 95% CI of the Se and Sp and considered statistically different from a random choice those that did not overlap 50%, which is the randomness region.

Cohen’s Kappa was used to measure the agreement of the two methods (sniffer dog and RAD) to screen people. Results were interpreted as follows: values ≤ 0 as indicating no agreement, 0.01–0.20 as none to slight, 0.21–0.40 as fair, 0.41–0.60 as moderate, 0.61–0.80 as substantial, and 0.81–1.00 as almost perfect agreement^55^.

For all the analyses, a two-sided p < 0.05 was considered statistically significant.

### Ethical statement

We confirm that the procedures comply with national and EU legislation. Research was performed in accordance with the Declaration of Helsinki. The study was approved by the Animal Welfare Committee (OPBA) of the University of Milan (OPBA_06_2021). Before participating in the olfactory detection test, each dog owner gave informed written consent for using their dogs’ test results in research. Reporting of results follows the recommendations of the ARRIVE guidelines. Informed consent was given by each subject for publication of identifying images in an online-access publication.

## Supplementary Information

Supplementary Table 1.

Supplementary Table 2.

Supplementary Table 3.

Supplementary Table 4.

## Data Availability

The data that support the findings of this study are available from the project supervisor M.A. upon reasonable request.
