# Supplementary material for: Sniffer dogs performance is stable over time in detecting COVID-19 positive samples and agrees with the rapid antigen test in the field
Source: Sci Rep. 2023 Mar 5;13:3679. doi: 10.1038/s41598-023-30897-1 (PMC9985821; doi:10.1038/s41598-023-30897-1)
Supplement: Supplementary file 1 — Supplementary Table 1. [file 41598_2023_30897_MOESM1_ESM.docx]

**Supplementary table 1.** Main characteristics of the samples employed during the test and retest phases in the laboratory

| Dog | Sample ID | RT_PCR result | Session | Run | Dog response | Participant gender | Participant age | Anti-COVID-19 vaccination |
| --- | --- | --- | --- | --- | --- | --- | --- | --- |
| helix | p_itlom040 | positive | 1 | 1 | 1 | m | 78 | no |
| helix | p_itlom071 | positive | 1 | 2 | 1 | f | 63 | no |
| helix | p_itlom074 | positive | 1 | 3 | 1 | f | 60 | no |
| helix | p_itlom073 | positive | 1 | 4 | 1 | f | 60 | no |
| helix | p_itlom87 | positive | 1 | 5 | 1 | m | 89 | no |
| helix | p_itlom119 | positive | 2 | 1 | 1 | f | 44 | no |
| helix | p_itlom135 | positive | 2 | 2 | 1 | m | 75 | no |
| helix | p_itlom133 | positive | 2 | 3 | 1 | f | 89 | no |
| helix | p_itlom129 | positive | 2 | 4 | 1 | f | 87 | yes |
| helix | p_itlom128 | positive | 2 | 5 | 1 | m | 74 | yes |
| otto | p_itlom135 | positive | 1 | 1 | 1 | m | 75 | yes |
| otto | p_itlom133 | positive | 1 | 2 | 1 | f | 89 | no |
| otto | p_itlom129 | positive | 1 | 3 | 0 | f | 87 | yes |
| otto | p_itlom128 | positive | 1 | 4 | 1 | m | 74 | yes |
| otto | p_itlom122 | positive | 1 | 5 | 1 | m | 44 | no |
| otto | p_itlom73 | positive | 2 | 1 | 1 | f | 60 | no |
| otto | p_itlom71 | positive | 2 | 2 | 1 | f | 63 | no |
| otto | p_itlom126 | positive | 2 | 3 | 1 | m | 72 | yes |
| otto | p_itlom40 | positive | 2 | 4 | 1 | m | 78 | no |
| otto | p_itlom125 | positive | 2 | 5 | 1 | m | 68 | yes |
| nala | p_itlom121 | positive | 1 | 1 | 0 | f | 74 | no |
| nala | ts_1 | positive | 1 | 2 | 1 | m | 45 | yes |
| nala | p_itlom123 | positive | 1 | 3 | 1 | f | 73 | no |
| nala | p_itlom124 | positive | 1 | 4 | 1 | m | 80 | yes |
| nala | ts_2 | positive | 1 | 5 | 1 | f | 39 | yes |
| nala | mmsx | positive | 2 | 1 | 1 | m | 37 | yes |
| nala | ts_2sn | positive | 2 | 2 | 1 | f | 39 | yes |
| nala | sgsn | positive | 2 | 3 | 1 | f | 34 | yes |
| nala | ms01dx | positive | 2 | 4 | 1 | f | 28 | yes |
| nala | p_itlom155 | positive | 2 | 5 | 1 | m | 48 | no |
| helix | ieo 110 | negative | 1 | 1 | 1 | f | 61 | yes |
| helix | ieo23 | negative | 1 | 1 | 1 | f | 48 | yes |
| helix | ieo38 | negative | 1 | 1 | 1 | f | 57 | yes |
| helix | ieo77 | negative | 1 | 1 | 1 | f | 35 | yes |
| helix | ieo28 | negative | 1 | 1 | 1 | f | 50 | yes |
| helix | ieo50 | negative | 1 | 2 | 1 | f | 38 | yes |
| helix | ieo90 | negative | 1 | 2 | 1 | f | 51 | yes |
| helix | ieo107 | negative | 1 | 2 | 1 | f | 39 | yes |
| helix | ieo136 | negative | 1 | 2 | 1 | m | 72 | yes |
| helix | ieo152 | negative | 1 | 2 | 1 | f | 45 | no |
| helix | ieo4 | negative | 1 | 3 | 1 | f | 60 | yes |
| helix | ieo16 | negative | 1 | 3 | 1 | f | 57 | yes |
| helix | ieo26 | negative | 1 | 3 | 1 | f | 67 | no |
| helix | ieo15 | negative | 1 | 3 | 1 | m | 66 | yes |
| helix | ieo150 | negative | 1 | 3 | 1 | f | 38 | yes |
| helix | ieo126 | negative | 1 | 4 | 1 | f | 67 | no |
| helix | ieo155 | negative | 1 | 4 | 1 | f | 54 | yes |
| helix | ieo129 | negative | 1 | 4 | 1 | f | 28 | yes |
| helix | ieo173 | negative | 1 | 4 | 1 | f | 53 | yes |
| helix | ieo22 | negative | 1 | 4 | 1 | m | 56 | yes |
| helix | ieo182 | negative | 1 | 5 | 1 | f | 65 | yes |
| helix | ieo149 | negative | 1 | 5 | 1 | f | 38 | yes |
| helix | ieo119 | negative | 1 | 5 | 1 | f | 40 | yes |
| helix | ieo141 | negative | 1 | 5 | 1 | f | 47 | yes |
| helix | ieo151 | negative | 1 | 5 | 1 | m | 60 | yes |
| helix | ieo46 | negative | 2 | 1 | 1 | f | 56 | yes |
| helix | ieo32 | negative | 2 | 1 | 1 | f | 50 | yes |
| helix | ieo34 | negative | 2 | 1 | 1 | f | 56 | yes |
| helix | ieo39 | negative | 2 | 1 | 1 | m | 63 | yes |
| helix | ieo36 | negative | 2 | 1 | 1 | m | 51 | no |
| helix | ieo59 | negative | 2 | 2 | 1 | m | 61 | no |
| helix | ieo84 | negative | 2 | 2 | 1 | f | 63 | yes |
| helix | ieo60 | negative | 2 | 2 | 1 | f | 28 | yes |
| helix | ieo47 | negative | 2 | 2 | 1 | f | 45 | yes |
| helix | ieo89 | negative | 2 | 2 | 1 | m | 59 | no |
| helix | ieo74 | negative | 2 | 3 | 1 | m | 50 | yes |
| helix | ieo73 | negative | 2 | 3 | 1 | m | 61 | yes |
| helix | ieo97 | negative | 2 | 3 | 1 | f | 69 | yes |
| helix | ieo40 | negative | 2 | 3 | 1 | f | 33 | yes |
| helix | ieo61 | negative | 2 | 3 | 1 | m | 57 | no |
| helix | ieo98 | negative | 2 | 4 | 1 | f | 73 | yes |
| helix | ieo44 | negative | 2 | 4 | 1 | m | 43 | yes |
| helix | ieo30 | negative | 2 | 4 | 1 | f | 55 | yes |
| helix | ieo27 | negative | 2 | 4 | 1 | f | 76 | yes |
| helix | ieo75 | negative | 2 | 4 | 1 | m | 32 | yes |
| helix | ieo33 | negative | 2 | 5 | 1 | f | 46 | no |
| helix | ieo29 | negative | 2 | 5 | 1 | m | 70 | yes |
| helix | ieo88 | negative | 2 | 5 | 1 | f | 50 | yes |
| helix | ieo11 | negative | 2 | 5 | 1 | m | 50 | yes |
| helix | ieo10 | negative | 2 | 5 | 1 | f | 61 | yes |
| otto | ieo222 | negative | 1 | 1 | 1 | m | 24 | yes |
| otto | ieo36 | negative | 1 | 1 | 1 | m | 51 | no |
| otto | ieo225 | negative | 1 | 1 | 1 | m | 56 | yes |
| otto | ieo195 | negative | 1 | 1 | 1 | f | 48 | yes |
| otto | ieo29 | negative | 1 | 1 | 1 | m | 70 | yes |
| otto | ieo211 | negative | 1 | 2 | 1 | f | 59 | yes |
| otto | ieo234 | negative | 1 | 2 | 1 | m | 70 | yes |
| otto | ieo27 | negative | 1 | 2 | 1 | f | 76 | yes |
| otto | ieo193 | negative | 1 | 2 | 1 | f | 55 | yes |
| otto | ieo189 | negative | 1 | 2 | 1 | f | 64 | yes |
| otto | ieo46 | negative | 1 | 3 | 1 | f | 56 | yes |
| otto | ieo187 | negative | 1 | 3 | 0 | f | 50 | yes |
| otto | ieo233 | negative | 1 | 3 | 1 | f | 52 | yes |
| otto | ieo216 | negative | 1 | 3 | 1 | f | 63 | yes |
| otto | ieo220 | negative | 1 | 3 | 1 | f | 55 | no |
| otto | ieo213 | negative | 1 | 4 | 1 | m | 70 | yes |
| otto | ieo223 | negative | 1 | 4 | 1 | f | 34 | no |
| otto | ieo244 | negative | 1 | 4 | 1 | f | 41 | yes |
| otto | ieo60 | negative | 1 | 4 | 1 | f | 28 | yes |
| otto | ieo44 | negative | 1 | 4 | 1 | m | 43 | yes |
| otto | ieo197 | negative | 1 | 5 | 1 | m | 64 | yes |
| otto | ieo190 | negative | 1 | 5 | 1 | m | 46 | yes |
| otto | ieo196 | negative | 1 | 5 | 1 | f | 46 | yes |
| otto | ieo47 | negative | 1 | 5 | 1 | f | 45 | yes |
| otto | ieo239 | negative | 1 | 5 | 1 | f | 50 | yes |
| otto | ieo89 | negative | 2 | 1 | 1 | m | 59 | no |
| otto | ieo215 | negative | 2 | 1 | 1 | f | 22 | yes |
| otto | ieo76 | negative | 2 | 1 | 1 | m | 68 | yes |
| otto | ieo32 | negative | 2 | 1 | 1 | f | 50 | yes |
| otto | ieo34 | negative | 2 | 1 | 1 | f | 56 | yes |
| otto | ieo9 | negative | 2 | 2 | 1 | f | 61 | yes |
| otto | ieo81 | negative | 2 | 2 | 1 | f | 71 | yes |
| otto | ieo129 | negative | 2 | 2 | 1 | f | 28 | yes |
| otto | ieo33 | negative | 2 | 2 | 1 | f | 46 | no |
| otto | ieo10 | negative | 2 | 2 | 1 | f | 61 | yes |
| otto | ieo37 | negative | 2 | 3 | 1 | m | 60 | yes |
| otto | ieo40 | negative | 2 | 3 | 1 | f | 33 | yes |
| otto | ieo44 | negative | 2 | 3 | 1 | m | 43 | yes |
| otto | ieo7 | negative | 2 | 3 | 1 | f | 36 | yes |
| otto | ieo6 | negative | 2 | 3 | 1 | f | 58 | no |
| otto | ieo85 | negative | 2 | 4 | 1 | f | 52 | yes |
| otto | ieo43 | negative | 2 | 4 | 1 | f | 50 | yes |
| otto | ieo8 | negative | 2 | 4 | 1 | f | 71 | yes |
| otto | ieo35 | negative | 2 | 4 | 1 | m | 51 | yes |
| otto | ieo50 | negative | 2 | 4 | 1 | f | 38 | yes |
| otto | ieo27 | negative | 2 | 5 | 1 | f | 76 | yes |
| otto | ieo43 | negative | 2 | 5 | 1 | f | 50 | yes |
| otto | ieo45 | negative | 2 | 5 | 1 | f | 45 | yes |
| otto | ieo41 | negative | 2 | 5 | 1 | f | 69 | yes |
| otto | ieo46 | negative | 2 | 5 | 1 | f | 56 | yes |
| nala | ieo170 | negative | 1 | 1 | 0 | f | 66 | yes |
| nala | ieo129 | negative | 1 | 1 | 1 | f | 28 | yes |
| nala | ieo108 | negative | 1 | 1 | 1 | f | 40 | yes |
| nala | ieo182 | negative | 1 | 1 | 1 | f | 65 | yes |
| nala | ieo150 | negative | 1 | 1 | 1 | m | 57 | yes |
| nala | ieo113 | negative | 1 | 2 | 1 | f | 49 | yes |
| nala | ieo131 | negative | 1 | 2 | 1 | f | 50 | yes |
| nala | ieo156 | negative | 1 | 2 | 1 | m | 51 | yes |
| nala | ieo125 | negative | 1 | 2 | 1 | f | 20 | yes |
| nala | ieo116 | negative | 1 | 2 | 1 | f | 49 | yes |
| nala | ieo168 | negative | 1 | 3 | 1 | f | 46 | yes |
| nala | ieo106 | negative | 1 | 3 | 1 | m | 72 | yes |
| nala | ieo151 | negative | 1 | 3 | 1 | m | 60 | yes |
| nala | ieo149 | negative | 1 | 3 | 1 | f | 38 | yes |
| nala | ieo155 | negative | 1 | 3 | 1 | f | 54 | yes |
| nala | ieo177 | negative | 1 | 4 | 1 | f | 40 | yes |
| nala | ieo105 | negative | 1 | 4 | 1 | f | 53 | no |
| nala | ieo173 | negative | 1 | 4 | 1 | f | 53 | yes |
| nala | ieo141 | negative | 1 | 4 | 1 | f | 47 | yes |
| nala | ieo118 | negative | 1 | 4 | 1 | f | 44 | yes |
| nala | ieo120 | negative | 1 | 5 | 1 | f | 52 | yes |
| nala | ieo136 | negative | 1 | 5 | 1 | m | 72 | yes |
| nala | ieo152 | negative | 1 | 5 | 1 | f | 45 | no |
| nala | ieo126 | negative | 1 | 5 | 1 | m | 68 | yes |
| nala | ieo107 | negative | 1 | 5 | 1 | f | 39 | yes |
| nala | l148 | negative | 2 | 1 | 1 | f | 70 | yes |
| nala | l142 | negative | 2 | 1 | 1 | f | 45 | no |
| nala | l138 | negative | 2 | 1 | 1 | f | 54 | yes |
| nala | l156 | negative | 2 | 1 | 1 | f | 24 | no |
| nala | l267 | negative | 2 | 1 | 1 | f | 42 | no |
| nala | l179 | negative | 2 | 2 | 1 | f | 40 | no |
| nala | l192 | negative | 2 | 2 | 1 | m | 20 | no |
| nala | l230 | negative | 2 | 2 | 1 | f | 47 | no |
| nala | l153 | negative | 2 | 2 | 1 | f | 30 | yes |
| nala | l261 | negative | 2 | 2 | 1 | m | 43 | no |
| nala | l312 | negative | 2 | 3 | 1 | f | 22 | no |
| nala | l295 | negative | 2 | 3 | 1 | m | 18 | no |
| nala | l197 | negative | 2 | 3 | 1 | f | 55 | yes |
| nala | l140 | negative | 2 | 3 | 1 | m | 50 | no |
| nala | l321 | negative | 2 | 3 | 1 | m | 61 | yes |
| nala | l276 | negative | 2 | 4 | 1 | f | 50 | yes |
| nala | l268 | negative | 2 | 4 | 1 | f | 45 | no |
| nala | l173 | negative | 2 | 4 | 1 | m | 51 | no |
| nala | ieo4 | negative | 2 | 4 | 1 | f | 60 | yes |
| nala | ieo22 | negative | 2 | 4 | 1 | m | 56 | yes |
| nala | ieo45 | negative | 2 | 5 | 1 | f | 45 | yes |
| nala | ieo90 | negative | 2 | 5 | 1 | f | 51 | yes |
| nala | ieo28 | negative | 2 | 5 | 1 | f | 50 | yes |
| nala | ieo38 | negative | 2 | 5 | 1 | f | 57 | yes |
| nala | ieo16 | negative | 2 | 5 | 1 | f | 57 | yes |
|  |  |  |  |  |  |  |  |  |
| helix | p_itlom121 | positive | 1 | 1 | 0 | f | 74 | no |
| helix | p_itlom123 | positive | 1 | 2 | 1 | f | 73 | no |
| helix | p_itlom124 | positive | 1 | 3 | 1 | m | 80 | yes |
| helix | ts_2 | positive | 1 | 4 | 1 | f | 39 | yes |
| helix | ts_1 | positive | 1 | 5 | 1 | m | 45 | yes |
| helix | p_itlom150 | positive | 2 | 1 | 1 | f | 81 | no |
| helix | p_itlom139 | positive | 2 | 2 | 0 | m | 85 | yes |
| helix | p_itlom112 | positive | 2 | 3 | 1 | m | 76 | yes |
| helix | p_itlom113 | positive | 2 | 4 | 0 | f | 69 | yes |
| helix | l233 | positive | 2 | 5 | 0 | f | 29 | no |
| otto | ts_1 | positive | 1 | 1 | 1 | m | 45 | yes |
| otto | p_itlom151 | positive | 1 | 2 | 0 | m | 61 | yes |
| otto | p_itlom145 | positive | 1 | 3 | 1 | m | 85 | yes |
| otto | p_itlom144 | positive | 1 | 4 | 1 | f | 82 | yes |
| otto | p_itlom148 | positive | 1 | 5 | 1 | f | 71 | yes |
| otto | p_itlom155 | positive | 2 | 1 | 1 | m | 48 | no |
| otto | p_itlom146 | positive | 2 | 2 | 1 | m | 83 | yes |
| otto | p_itlom147 | positive | 2 | 3 | 1 | m | 72 | yes |
| otto | p_itlom149 | positive | 2 | 4 | 1 | f | 95 | yes |
| otto | ts_2 | positive | 2 | 5 | 1 | f | 39 | yes |
| nala | p_itlom157 | positive | 1 | 1 | 1 | m | 84 | yes |
| nala | p_itlom164 | positive | 1 | 2 | 1 | f | 82 | yes |
| nala | p_itlom159 | positive | 1 | 3 | 1 | m | 74 | yes |
| nala | p_itlom162 | positive | 1 | 4 | 1 | m | 75 | yes |
| nala | p_itlom158 | positive | 1 | 5 | 1 | m | 69 | yes |
| nala | p_itlom156 | positive | 2 | 1 | 1 | f | 87 | no |
| nala | p_itlom163 | positive | 2 | 2 | 1 | m | 79 | yes |
| nala | p_itlom161 | positive | 2 | 3 | 1 | m | 73 | yes |
| nala | p_itlom165 | positive | 2 | 4 | 1 | f | 72 | no |
| nala | p_itlom160 | positive | 2 | 5 | 1 | m | 86 | no |
| helix | ieo124 | negative | 1 | 1 | 1 | f | 67 | yes |
| helix | ieo100 | negative | 1 | 1 | 1 | m | 58 | yes |
| helix | ieo99 | negative | 1 | 1 | 0 | f | 38 | yes |
| helix | ieo172 | negative | 1 | 1 | 1 | f | 43 | yes |
| helix | ieo135 | negative | 1 | 1 | 1 | f | 60 | yes |
| helix | ieo132 | negative | 1 | 2 | 1 | f | 40 | yes |
| helix | ieo82 | negative | 1 | 2 | 1 | f | 46 | yes |
| helix | ieo150 | negative | 1 | 2 | 1 | m | 57 | yes |
| helix | ieo179 | negative | 1 | 2 | 1 | f | 54 | yes |
| helix | ieo134 | negative | 1 | 2 | 1 | f | 51 | yes |
| helix | ieo122 | negative | 1 | 3 | 1 | m | 63 | yes |
| helix | ieo147 | negative | 1 | 3 | 1 | f | 34 | yes |
| helix | ieo101 | negative | 1 | 3 | 1 | f | 69 | yes |
| helix | ieo113 | negative | 1 | 3 | 1 | f | 49 | yes |
| helix | ieo92 | negative | 1 | 3 | 1 | f | 50 | yes |
| helix | ieo143 | negative | 1 | 4 | 1 | m | 71 | yes |
| helix | ieo174 | negative | 1 | 4 | 1 | f | 61 | yes |
| helix | ieo95 | negative | 1 | 4 | 1 | f | 60 | yes |
| helix | ieo96 | negative | 1 | 4 | 1 | f | 56 | yes |
| helix | ieo167 | negative | 1 | 4 | 1 | f | 36 | yes |
| helix | ieo102 | negative | 1 | 5 | 1 | f | 51 | no |
| helix | ieo138 | negative | 1 | 5 | 1 | m | 42 | yes |
| helix | ieo86 | negative | 1 | 5 | 1 | f | 54 | no |
| helix | ieo142 | negative | 1 | 5 | 1 | f | 69 | yes |
| helix | ieo87 | negative | 1 | 5 | 1 | f | 66 | yes |
| helix | l164 | negative | 2 | 1 | 1 | f | 55 | yes |
| helix | l26 | negative | 2 | 1 | 1 | f | 59 | yes |
| helix | l100 | negative | 2 | 1 | 1 | m | 50 | yes |
| helix | l158 | negative | 2 | 1 | 1 | f | 49 | no |
| helix | l64 | negative | 2 | 1 | 1 | m | 56 | yes |
| helix | l90 | negative | 2 | 2 | 1 | f | 62 | yes |
| helix | l84 | negative | 2 | 2 | 1 | f | 28 | no |
| helix | l129 | negative | 2 | 2 | 1 | m | 45 | no |
| helix | l326 | negative | 2 | 2 | 1 | f | 64 | yes |
| helix | l274 | negative | 2 | 2 | 0 | f | 52 | yes |
| helix | l177 | negative | 2 | 3 | 1 | f | 22 | no |
| helix | l227 | negative | 2 | 3 | 1 | m | 49 | no |
| helix | l127 | negative | 2 | 3 | 1 | f | 43 | yes |
| helix | l287 | negative | 2 | 3 | 1 | f | 33 | no |
| helix | l168 | negative | 2 | 3 | 1 | f | 35 | yes |
| helix | l284 | negative | 2 | 4 | 1 | f | 41 | no |
| helix | l188 | negative | 2 | 4 | 1 | m | 53 | no |
| helix | l272 | negative | 2 | 4 | 1 | f | 48 | yes |
| helix | l75 | negative | 2 | 4 | 0 | m | 60 | no |
| helix | l286 | negative | 2 | 4 | 1 | f | 45 | no |
| helix | l174 | negative | 2 | 5 | 0 | f | 32 | yes |
| helix | l86 | negative | 2 | 5 | 1 | m | 49 | yes |
| helix | l167 | negative | 2 | 5 | 1 | f | 58 | yes |
| helix | l189 | negative | 2 | 5 | 1 | f | 65 | yes |
| helix | l159 | negative | 2 | 5 | 1 | f | 39 | yes |
| otto | ieo252 | negative | 1 | 1 | 1 | f | 20 | yes |
| otto | ieo253 | negative | 1 | 1 | 1 | f | 64 | yes |
| otto | ieo389 | negative | 1 | 1 | 1 | m | 64 | yes |
| otto | ieo379 | negative | 1 | 1 | 1 | m | 33 | yes |
| otto | ieo251 | negative | 1 | 1 | 1 | f | 28 | yes |
| otto | ieo382 | negative | 1 | 2 | 0 | f | 31 | yes |
| otto | ieo385 | negative | 1 | 2 | 1 | f | 28 | yes |
| otto | ieo388 | negative | 1 | 2 | 1 | m | 52 | yes |
| otto | ieo381 | negative | 1 | 2 | 1 | f | 37 | yes |
| otto | ieo380 | negative | 1 | 2 | 1 | m | 50 | yes |
| otto | ieo250 | negative | 1 | 3 | 1 | m | 57 | yes |
| otto | ieo254 | negative | 1 | 3 | 1 | f | 44 | yes |
| otto | ieo249 | negative | 1 | 3 | 1 | m | 38 | no |
| otto | ieo391 | negative | 1 | 3 | 1 | f | 48 | yes |
| otto | ieo383 | negative | 1 | 3 | 1 | f | 48 | yes |
| otto | ieo112 | negative | 1 | 4 | 1 | f | 50 | no |
| otto | ieo390 | negative | 1 | 4 | 1 | f | 49 | yes |
| otto | ieo384 | negative | 1 | 4 | 1 | m | 44 | yes |
| otto | ieo113 | negative | 1 | 4 | 1 | f | 49 | yes |
| otto | ieo131 | negative | 1 | 4 | 1 | f | 50 | yes |
| otto | ieo176 | negative | 1 | 5 | 1 | f | 42 | yes |
| otto | ieo163 | negative | 1 | 5 | 1 | f | 46 | yes |
| otto | ieo116 | negative | 1 | 5 | 1 | f | 49 | yes |
| otto | ieo125 | negative | 1 | 5 | 1 | f | 20 | yes |
| otto | ieo115 | negative | 1 | 5 | 1 | f | 62 | yes |
| otto | ieo106 | negative | 2 | 1 | 1 | m | 72 | yes |
| otto | ieo1 | negative | 2 | 1 | 1 | f | 67 | yes |
| otto | ieo182 | negative | 2 | 1 | 1 | f | 65 | yes |
| otto | ieo77 | negative | 2 | 1 | 1 | f | 35 | yes |
| otto | ieo9o | negative | 2 | 1 | 1 | f | 51 | yes |
| otto | ieo149 | negative | 2 | 2 | 1 | f | 38 | yes |
| otto | ieo58 | negative | 2 | 2 | 1 | m | 40 | yes |
| otto | ieo177 | negative | 2 | 2 | 1 | f | 40 | yes |
| otto | ieo133 | negative | 2 | 2 | 1 | f | 47 | yes |
| otto | ieo136 | negative | 2 | 2 | 1 | m | 72 | yes |
| otto | ieo169 | negative | 2 | 3 | 1 | m | 57 | no |
| otto | ieo110 | negative | 2 | 3 | 1 | m | 51 | yes |
| otto | ieo126 | negative | 2 | 3 | 1 | m | 68 | yes |
| otto | ieo139 | negative | 2 | 3 | 1 | f | 60 | yes |
| otto | ieo104 | negative | 2 | 3 | 1 | m | 64 | yes |
| otto | ieo160 | negative | 2 | 4 | 1 | f | 43 | no |
| otto | ieo161 | negative | 2 | 4 | 1 | f | 32 | yes |
| otto | ieo26 | negative | 2 | 4 | 1 | m | 67 | no |
| otto | ieo107 | negative | 2 | 4 | 1 | f | 39 | yes |
| otto | ieo108 | negative | 2 | 4 | 1 | f | 40 | yes |
| otto | ieo120 | negative | 2 | 5 | 1 | f | 52 | yes |
| otto | ieo170 | negative | 2 | 5 | 1 | f | 66 | yes |
| otto | ieo128 | negative | 2 | 5 | 1 | f | 64 | yes |
| otto | ieo118 | negative | 2 | 5 | 1 | f | 44 | yes |
| otto | ieo173 | negative | 2 | 5 | 1 | f | 53 | yes |
| nala | ieo111 | negative | 1 | 1 | 1 | f | 56 | yes |
| nala | b95 | negative | 1 | 1 | 1 | f | 36 | yes |
| nala | b28 | negative | 1 | 1 | 1 | m | 40 | yes |
| nala | b58 | negative | 1 | 1 | 1 | m | 30 | yes |
| nala | b78 | negative | 1 | 1 | 1 | m | 26 | yes |
| nala | l136 | negative | 1 | 2 | 1 | m | 45 | yes |
| nala | b63 | negative | 1 | 2 | 1 | f | 22 | yes |
| nala | b80 | negative | 1 | 2 | 1 | m | 38 | yes |
| nala | ieo127 | negative | 1 | 2 | 1 | f | 73 | yes |
| nala | b69 | negative | 1 | 2 | 1 | f | 25 | yes |
| nala | l288 | negative | 1 | 3 | 1 | m | 29 | yes |
| nala | b60 | negative | 1 | 3 | 1 | m | 29 | yes |
| nala | ieo25 | negative | 1 | 3 | 1 | m | 57 | yes |
| nala | b92 | negative | 1 | 3 | 1 | m | 34 | yes |
| nala | ieo76 | negative | 1 | 3 | 1 | m | 68 | yes |
| nala | l280 | negative | 1 | 4 | 1 | m | 55 | yes |
| nala | ieo117 | negative | 1 | 4 | 1 | m | 71 | yes |
| nala | b75 | negative | 1 | 4 | 1 | m | 32 | yes |
| nala | b94 | negative | 1 | 4 | 1 | m | 43 | yes |
| nala | b13 | negative | 1 | 4 | 1 | m | 27 | yes |
| nala | b11 | negative | 1 | 5 | 1 | m | 43 | yes |
| nala | b16 | negative | 1 | 5 | 1 | m | 23 | yes |
| nala | b73 | negative | 1 | 5 | 1 | m | 41 | yes |
| nala | b51 | negative | 1 | 5 | 1 | m | 43 | yes |
| nala | ieo123 | negative | 1 | 5 | 1 | m | 69 | yes |
| nala | ieo37 | negative | 2 | 1 | 1 | m | 60 | yes |
| nala | ieo119 | negative | 2 | 1 | 1 | f | 40 | yes |
| nala | l317 | negative | 2 | 1 | 1 | m | 37 | yes |
| nala | ieo171 | negative | 2 | 1 | 1 | m | 52 | yes |
| nala | l155 | negative | 2 | 1 | 1 | m | 21 | no |
| nala | ieo167 | negative | 2 | 2 | 1 | f | 36 | yes |
| nala | ieo169 | negative | 2 | 2 | 1 | m | 57 | no |
| nala | b72 | negative | 2 | 2 | 1 | f | 34 | yes |
| nala | ieo32 | negative | 2 | 2 | 1 | f | 50 | yes |
| nala | ieo133 | negative | 2 | 2 | 1 | f | 47 | yes |
| nala | l283 | negative | 2 | 3 | 1 | m | 53 | yes |
| nala | ieo40 | negative | 2 | 3 | 1 | f | 33 | yes |
| nala | ieo43 | negative | 2 | 3 | 1 | f | 50 | yes |
| nala | ieo33 | negative | 2 | 3 | 1 | f | 46 | no |
| nala | ieo80 | negative | 2 | 3 | 1 | m | 70 | yes |
| nala | ieo21 | negative | 2 | 4 | 1 | m | 75 | yes |
| nala | ieo10 | negative | 2 | 4 | 1 | f | 61 | yes |
| nala | ieo35 | negative | 2 | 4 | 1 | m | 51 | yes |
| nala | b20 | negative | 2 | 4 | 1 | m | 23 | yes |
| nala | b77 | negative | 2 | 4 | 1 | m | 22 | yes |
| nala | ieo89 | negative | 2 | 5 | 1 | m | 59 | no |
| nala | ieo81 | negative | 2 | 5 | 1 | f | 71 | yes |
| nala | b76 | negative | 2 | 5 | 1 | f | 27 | yes |
| nala | ieo77 | negative | 2 | 5 | 1 | f | 35 | yes |
| nala | b61 | negative | 2 | 5 | 1 | m | 30 | yes |
