# Supplementary material for: Sniffer dogs performance is stable over time in detecting COVID-19 positive samples and agrees with the rapid antigen test in the field
Source: Sci Rep. 2023 Mar 5;13:3679. doi: 10.1038/s41598-023-30897-1 (PMC9985821; doi:10.1038/s41598-023-30897-1)
Supplement: Supplementary file 3 — Supplementary Table 3. [file 41598_2023_30897_MOESM3_ESM.docx]

**Supplementary table 3.** Summary of results from volunteers who tested negative on the rapid antigen test (RAD) and were sniffed by the sniffer dogs in the field work, grouped based on being sniffed by a single dog (one) or multiple dogs (multiple).

| **Progressive number per group** | **Volunteer code** | **Group: Number of sniffer dogs on the volunteer (one *vs* multiple)** | **Volunteer gender** | **Dog** | **Antigen test** | **Sniffer dog** | **Notes** |
| --- | --- | --- | --- | --- | --- | --- | --- |
| 1 | dd | one | F | NALA | negative | negative | symptoms |
| 2 | fnirzo | one | M | NALA | negative | negative |  |
| 3 | ftu3 | one | M | HOPE | negative | negative |  |
| 4 | ftd | one | F | HOPE | negative | negative |  |
| 5 | dmzd | one | M | HOPE | negative | negative |  |
| 6 | fnrzo | one | M | HOPE | negative | negative |  |
| 7 | fnu | one | M | HOPE | negative | positive |  |
| 8 | fmbno | one | M | HOPE | negative | negative |  |
| 9 | fmpbano | one | M | HOPE | negative | negative |  |
| 10 | fmanzo | one | M | HOPE | negative | negative |  |
| 11 | fmad2 | one | F | HOPE | negative | negative |  |
| 12 | fnirzina | one | F | HOPE | negative | negative |  |
| 13 | fnid5 | one | F | HOPE | negative | negative |  |
| 14 | ftd2 | one | F | HOPE | negative | negative |  |
| 15 | fnirzo3 | one | M | HOPE | negative | negative |  |
| 16 | fniu3 | one | M | HOPE | negative | negative |  |
| 17 | fnid9 | one | F | HOPE | negative | negative |  |
| 18 | fnid10 | one | F | HOPE | negative | negative |  |
| 19 | fniu4 | one | M | HOPE | negative | negative |  |
| 20 | fniu6 | one | M | HOPE | negative | negative |  |
| 21 | fnid13 | one | F | HOPE | negative | negative |  |
| 22 | dcs | one | F | HOPE | negative | negative |  |
| 23 | dra | one | F | HOPE | negative | negative |  |
| 24 | ftanzo2 | one | M | NIM | negative | negative |  |
| 25 | ftanzo | one | M | NIM | negative | negative |  |
| 26 | fnpp | one | M | NIM | negative | negative |  |
| 27 | fnmm | one | F | NIM | negative | negative |  |
| 28 | fnfgl | one | F | NIM | negative | negative |  |
| 29 | fnrzo2 | one | M | NIM | negative | negative |  |
| 30 | fnid15 | one | F | NIM | negative | negative | symptoms |
| 31 | fnrzo3 | one | M | CHAOS | negative | negative |  |
| 1 | dqa | multiple | F | NALA | negative | negative |  |
|  |  |  |  | IRIS | negative | negative |  |
|  |  |  |  | NIM | negative | negative |  |
|  |  |  |  | CHAOS | negative | negative |  |
|  |  |  |  | HOPE | negative | negative |  |
| 2 | dalb | multiple | F | NALA | negative | negative |  |
|  |  |  |  | HOPE | negative | negative |  |
| 3 | fnifu | multiple | M | NALA | negative | negative |  |
|  |  |  |  | HOPE | negative | negative |  |
| 4 | fnifd | multiple | F | NALA | negative | negative |  |
|  |  |  |  | HOPE | negative | negative |  |
| 5 | drev | multiple | F | HOPE | negative | negative |  |
|  |  |  |  | IRIS | negative | negative |  |
|  |  |  |  | NIM | negative | negative |  |
|  |  |  |  | CHAOS | negative | negative |  |
| 6 | dce | multiple | M | NIM | negative | negative |  |
|  |  |  |  | NALA | negative | negative |  |
|  |  |  |  | HOPE | negative | negative |  |
| 7 | dpl | multiple | F | NALA | negative | negative |  |
|  |  |  |  | HOPE | negative | negative |  |
|  |  |  |  | NIM | negative | negative |  |
| 8 | fnirzo2 | multiple | M | HOPE | negative | negative |  |
|  |  |  |  | NALA | negative | negative |  |
| 9 | fnirza | multiple | F | NALA | negative | negative |  |
|  |  |  |  | HOPE | negative | negative |  |
| 10 | dgr | multiple | F | IRIS | negative | negative |  |
|  |  |  |  | CHAOS | negative | negative |  |
| 11 | dmr | multiple | M | IRIS | negative | negative |  |
|  |  |  |  | CHAOS | negative | negative |  |
| 12 | dcg | multiple | M | IRIS | negative | negative |  |
|  |  |  |  | CHAOS | negative | negative |  |
| 13 | dsa | multiple | F | IRIS | negative | negative |  |
|  |  |  |  | CHAOS | negative | negative |  |
| 14 | fnid3 | multiple | F | NALA | negative | negative |  |
|  |  |  |  | HOPE | negative | negative |  |
| 15 | fnid4 | multiple | F | NALA | negative | negative |  |
|  |  |  |  | HOPE | negative | negative |  |
| 16 | fnim | multiple | F | NIM | negative | positive |  |
|  |  |  |  | HOPE | negative | negative |  |
| 17 | fnid11 | multiple | F | HOPE | negative | positive | symptoms |
|  |  |  |  | NIM | negative | positive |  |
| 18 | ftu | multiple | M | NALA | negative | negative |  |
|  |  |  |  | HOPE | negative | negative |  |
| 19 | dpa | multiple | M | NALA | negative | negative |  |
|  |  |  |  | HOPE | negative | negative |  |
|  |  |  |  | NIM | negative | negative |  |
| 20 | fmabno2 | multiple | M | HOPE | negative | negative |  |
|  |  |  |  | IRIS | negative | negative |  |
| 21 | fmad | multiple | F | HOPE | negative | negative | symptoms |
|  |  |  |  | IRIS | negative | negative |  |
| 22 | fmanza2 | multiple | F | HOPE | negative | negative |  |
|  |  |  |  | IRIS | negative | positive |  |
| 23 | fmanzo2 | multiple | M | HOPE | negative | negative |  |
|  |  |  |  | IRIS | negative | negative |  |
| 24 | fmarzo | multiple | M | HOPE | negative | negative |  |
|  |  |  |  | IRIS | negative | negative |  |
| 25 | fnid | multiple | F | NALA | negative | negative |  |
|  |  |  |  | HOPE | negative | negative |  |
| 26 | fnirza2 | multiple | F | NALA | negative | negative |  |
|  |  |  |  | HOPE | negative | negative |  |
| 27 | ftrzo | multiple | M | NALA | negative | negative |  |
|  |  |  |  | HOPE | negative | negative |  |
