# Supplementary material for: Sniffer dogs performance is stable over time in detecting COVID-19 positive samples and agrees with the rapid antigen test in the field
Source: Sci Rep. 2023 Mar 5;13:3679. doi: 10.1038/s41598-023-30897-1 (PMC9985821; doi:10.1038/s41598-023-30897-1)
Supplement: Supplementary file 4 — Supplementary Table 4. [file 41598_2023_30897_MOESM4_ESM.docx]

**Supplementary table 4**. Training of sniffer dogs for field work on volunteers

| **DOG** | **Volunteer code** | **Volunteer gender** | **Antigen test** | **Sniffer dog** | **Notes** |
| --- | --- | --- | --- | --- | --- |
| NALA | din | M | positive | positive | no symptoms |
| NALA | ft | M | positive | positive | no symptoms |
| NALA | ft1 | M | positive | positive | no symptoms |
| NALA | din | M | negative | negative | symptoms |
| NALA | ft2 | F | negative | negative |  |
| NALA | ft3 | M | negative | negative |  |
| NALA | ft4 | M | positive | positive | no symptoms |
| NALA | ft5 | M | negative | negative |  |
| NALA | ft6 | F | positive | positive | no symptoms |
| NALA | ft7 | M | negative | negative |  |
| NALA | fn | M | negative | negative |  |
| NALA | fn1 | M | negative | negative |  |
| NALA | fn2 | M | negative | negative |  |
| NALA | fn3 | F | negative | negative |  |
| NALA | fn4 | M | negative | negative |  |
| NALA | ft8 | F | negative | negative |  |
| NALA | ft9 | F | negative | negative |  |
| NALA | ft10 | M | negative | negative |  |
| NALA | ft11 | M | positive | positive | no symptoms |
| NALA | ft12 | M | positive | positive | no symptoms |
| NALA | ft13 | M | negative | negative | symptoms |
| NALA | ft14 | M | positive | positive | no symptoms |
| NALA | ft15 | M | positive | positive |  |
| NALA | ft16 | F | negative | negative |  |
| NALA | fni | M | negative | negative |  |
| NALA | fni1 | M | positive | positive | no symptoms |
| NALA | fni2 | M | positive | positive | no symptoms |
| NALA | fni3 | M | negative | negative |  |
| NALA | fni4 | F | negative | negative |  |
| NALA | fni5 | F | negative | negative |  |
| NALA | fni6 | F | negative | negative |  |
| NALA | fni7 | F | negative | negative |  |
| NALA | fni8 | F | negative | negative |  |
| NALA | fni9 | M | negative | negative |  |
| NALA | fni10 | F | negative | negative |  |
| NALA | fni11 | F | positive | positive | no symptoms |
| NALA | fn5 | F | negative | negative |  |
| NALA | fn6 | F | positive | positive | no symptoms |
| NALA | ft17 | F | negative | negative |  |
| NALA | drfa | F | positive | positive | no symptoms |
| NALA | dnm | F | negative | negative |  |
| NALA | dnf | F | positive | positive | no symptoms |
| NALA | dnp | M | negative | negative |  |
| NALA | fni12 | F | positive | positive |  |
| NALA | fni13 | F | positive | positive | weak positive |
| NALA | fni14 | M | positive | positive | no symptoms |
| NALA | fni15 | M | negative | negative |  |
| NALA | fni16 | F | negative | negative |  |
| NALA | fni17 | F | positive | positive | no symptoms |
| NALA | fnis | F | positive | positive | no symptoms |
| NALA | fni18 | M | negative | negative |  |
| NALA | fni19 | M | positive | positive | no symptoms |
| NALA | fni20 | M | negative | negative |  |
| NALA | fni21 | M | negative | negative |  |
| NALA | fma | F | negative | negative | symptoms |
| NALA | fma1 | M | negative | negative |  |
| NALA | fma2 | F | positive | negative | no symptoms |
| NALA | fma3 | F | negative | negative |  |
| NALA | fma4 | F | negative | negative |  |
| NALA | fma5 | F | negative | negative |  |
| NALA | fni22 | F | negative | negative |  |
| NALA | fni23 | F | positive | negative | no symptoms |
| NALA | fni24 | M | negative | negative |  |
| NALA | fni25 | M | negative | negative |  |
| NALA | fni26 | M | negative | negative |  |
| NALA | fni27 | F | positive | positive | weak positive |
| NALA | fni28 | M | positive | positive | no symptoms |
| NALA | fni29 | M | positive | positive | no symptoms |
| NALA | fni30 | F | negative | negative |  |
| NALA | fni31 | F | negative | negative |  |
| NALA | fni32 | F | negative | negative |  |
| NALA | fni33 | F | negative | negative |  |
| NALA | fni34 | F | positive | positive | weak positive |
| NALA | fni35 | F | positive | positive | no symptoms |
| NALA | fni36 | M | negative | negative |  |
| NALA | fni37 | M | negative | negative |  |
| NALA | fni38 | F | negative | negative | symptoms |
| NALA | fni39 | F | negative | negative | symptoms |
| NALA | fni40 | F | negative | negative | symptoms |
| NALA | fni41 | M | negative | negative |  |
| NALA | ft18 | M | positive | negative | no symptoms |
| NALA | ft19 | F | negative | negative |  |
| NALA | ft20 | F | positive | positive | no symptoms |
| NALA | ft21 | M | negative | negative |  |
| NALA | fni42 | F | positive | negative | no symptoms |
| NALA | fni43 | F | negative | negative |  |
| NALA | fni44 | M | negative | positive |  |
| NALA | fni45 | F | positive | positive | no symptoms |
| HOPE | ft22 | M | positive | positive | no symptoms |
| HOPE | dzd | F | positive | positive |  |
| HOPE | dfzd | M | negative | positive |  |
| HOPE | fn7 | M | negative | negative |  |
| HOPE | fn8 | F | negative | negative |  |
| HOPE | fni46 | M | negative | negative |  |
| HOPE | fni47 | M | positive | positive | no symptoms |
| HOPE | fni48 | F | negative | negative |  |
| HOPE | fni49 | M | negative | negative |  |
| HOPE | fni50 | F | negative | negative |  |
| HOPE | fni51 | M | negative | negative |  |
| HOPE | fni52 | F | negative | negative |  |
| HOPE | fni53 | F | negative | negative |  |
| HOPE | fni54 | M | negative | negative |  |
| HOPE | fni55 | F | negative | negative |  |
| HOPE | drfa1 | F | positive | positive |  |
| HOPE | dnm1 | F | negative | negative |  |
| HOPE | dnf1 | F | positive | positive |  |
| HOPE | dnp1 | M | negative | negative |  |
| HOPE | fn56 | F | positive | positive |  |
| HOPE | fni57 | F | positive | positive | weak positive |
| HOPE | fni58 | M | negative | negative |  |
| HOPE | fni59 | F | positive | positive | no symptoms |
| HOPE | fnis1 | F | positive | positive | no symptoms |
| HOPE | fni60 | M | negative | negative |  |
| HOPE | fni61 | M | negative | negative |  |
| HOPE | fni62 | M | negative | negative |  |
| HOPE | fni63 | F | negative | negative |  |
| HOPE | fni64 | F | positive | positive | weak positive |
| HOPE | fni65 | M | positive | positive | no symptoms |
| HOPE | fni66 | M | positive | positive | no symptoms |
| HOPE | fni67 | M | positive | positive | no symptoms |
| HOPE | fni68 | F | negative | negative |  |
| HOPE | fni69 | F | negative | negative |  |
| HOPE | fni70 | F | negative | negative |  |
| HOPE | fni71 | F | positive | positive | weak positive |
| HOPE | fni72 | F | positive | positive | no symptoms |
| HOPE | fni73 | M | positive | positive | no symptoms |
| HOPE | fni74 | M | negative | negative |  |
| HOPE | fni75 | F | negative | negative | symptoms |
| HOPE | fni76 | F | negative | negative | symptoms |
| HOPE | fni77 | F | negative | negative | symptoms |
| HOPE | fni78 | M | negative | negative |  |
| HOPE | fni79 | M | negative | negative |  |
| HOPE | fni80 | F | negative | negative |  |
| HOPE | fni81 | F | negative | negative |  |
| HOPE | fni82 | F | negative | positive |  |
| HOPE | fni83 | M | positive | positive | no symptoms |
| HOPE | fni84 | M | negative | negative |  |
| HOPE | dna | M | negative | positive |  |
| HOPE | fni85 | M | positive | positive |  |
| HOPE | fni86 | M | positive | positive |  |
| IRIS | di | M | positive | positive | no symptoms |
| IRIS | ft23 | M | negative | negative |  |
| IRIS | ft24 | M | positive | positive | no symptoms |
| IRIS | ft25 | F | negative | negative |  |
| IRIS | ft26 | F | negative | negative |  |
| IRIS | ft27 | F | positive | positive | no symptoms |
| IRIS |  | M | positive | negative | no symptoms |
| IRIS | ft28 | F | negative | negative |  |
| IRIS | ft29 | F | negative | negative |  |
| IRIS | ft30 | M | negative | negative |  |
| IRIS | ft31 | M | positive | positive | no symptoms |
| IRIS | ft32 | M | positive | positive | no symptoms |
| IRIS | ft33 | M | negative | negative | symptoms |
| IRIS | ft34 | M | positive | positive | no symptoms |
| IRIS | ft25 | F | negative | negative |  |
| IRIS | fni87 | F | negative | negative |  |
| IRIS | fni88 | F | negative | negative |  |
| IRIS | fni89 | F | negative | negative |  |
| IRIS | fni90 | F | negative | negative |  |
| IRIS | fni91 | M | negative | negative |  |
| IRIS | fni92 | F | negative | negative |  |
| IRIS | fma6 | M | negative | negative |  |
| IRIS | fma7 | F | negative | negative |  |
| IRIS | fma8 | F | negative | negative | symptoms |
| IRIS | fma9 | M | negative | negative |  |
| IRIS | fma10 | F | positive | negative | no symptoms |
| IRIS | fma11 | F | negative | negative |  |
| IRIS | fma12 | F | negative | negative |  |
| IRIS | fma13 | M | positive | positive | weak positive |
| IRIS | fma14 | M | positive | positive | weak positive |
| IRIS | fma15 | M | positive | positive | weak positive |
| IRIS | fma16 | F | negative | negative |  |
| NIM | di1 | M | positive | positive | no symptoms |
| NIM | ft36 | F | negative | negative |  |
| NIM | ft37 | F | negative | negative |  |
| NIM | ft38 | F | negative | negative |  |
| NIM | ft39 | F | negative | positive |  |
| NIM | fni93 | F | negative | negative |  |
| NIM | fmag | F | negative | negative |  |
| NIM | fni94 | F | negative | negative |  |
| NIM | fni95 | M | negative | negative |  |
| NIM | fni96 | F | negative | negative |  |
| CHAOS | di2 | M | positive | positive | no symptoms |
| CHAOS | fmag1 | F | negative | negative |  |
| CHAOS | fnag2 | F | negative | negative |  |
| CHAOS | fmag3 | F | positive | positive |  |
| CHAOS | fmag4 | M | negative | negative |  |
| CHAOS | fmag5 | F | negative | negative |  |
| CHAOS | fn9 | M | negative | negative |  |
| CHAOS | fn10 | F | negative | negative |  |
| CHAOS | fn11 | F | positive | positive | no symptoms |
| CHAOS | fn12 | F | negative | negative |  |
